# Market forces influence helping behaviour in cooperatively breeding paper wasps

**DOI:** 10.1038/ncomms13750

**Published:** 2017-01-24

**Authors:** Lena Grinsted, Jeremy Field

**Affiliations:** 1 School of Life Sciences, University of Sussex, John Maynard Smith Building, Falmer, Brighton BN1 9QG, UK

## Abstract

Biological market theory is potentially useful for understanding helping behaviour in animal societies. It predicts that competition for trading partners will affect the value of commodities exchanged. It has gained empirical support in cooperative breeders, where subordinates help dominant breeders in exchange for group membership, but so far without considering one crucial aspect: outside options. We find support for a biological market in paper wasps, *Polistes dominula*. We first show that females have a choice of cooperative partners. Second, by manipulating entire subpopulations in the field, we increase the supply of outside options for subordinates, freeing up suitable nesting spots and providing additional nesting partners. We predicted that by intensifying competition for help, our manipulation would force dominants to accept a lower price for group membership. As expected, subordinates reduce their foraging effort following our treatments. We conclude that to accurately predict the amount of help provided, social units cannot be viewed in isolation: the surrounding market must also be considered.

## Abstract (web summary)

In cooperatively breeding species, subordinates help to raise the dominant breeders' offspring in return for benefits associated with group membership. Here, Grinsted and Field show that the amount of help provided by subordinate paper wasps depends on the availability of alternative nesting options, as predicted by biological market theory.

Economics has long been an inspiration to evolutionary biologists. Indeed, Darwin himself was influenced by the economic theories of Malthus and Adam Smith when formulating his theory of evolution^1^. More recently, market theory has been suggested as a useful framework for understanding cooperative interactions within and between species^2^,^3^,^4^,^5^,^6^,^7^. Biological market theory emphasizes the importance of partner choice and competition for cooperative partners, using the economic principles of supply and demand to predict the trade-value of commodities exchanged between trader classes^2^,^3^,^8^,^9^.

Cooperatively breeding societies, in which subordinate helpers care for the offspring of dominant breeders, seem to provide a natural application for market principles^2^,^3^,^10^,^11^. Helpers effectively exchange commodities such as provisioning and defence of the dominant breeder's (or breeding pair's) offspring in return for group membership^12^,^13^. Breeders thus gain help with rearing their offspring, while subordinates may gain direct or indirect fitness benefits, for example via inheritance of the breeding position or by helping a relative^13^,^14^. However, while the idea that supply and demand affect the exchange of commodities between dominants and subordinates has been explored in several cooperative mammals^15^,^16^,^17^, outside options that should affect competition for partners have not been taken into account. Grooming has been the focus of most such studies, where dominant breeders either appear to ‘pay-for-help' (moustached tamarins, *Saguinus mystax*^15^, and common marmosets, *Callithrix jacchus*^16^) or subordinates appear to ‘pay-to-stay'^18^ (meerkats, *Suricata suricatta*^17^). These studies report observational data consistent with predictions from biological market theory: within groups with more subordinates, breeders either groom subordinates less (that is, pay less) in exchange for their help^15^,^16^, or subordinates groom dominants more (that is, pay more) for group membership^17^. In both cases, the greater supply of grooming as a commodity in larger groups decreases its trade value, with each additional helper having *per capita* less value to the breeder(s).

However, a key feature of biological market theory has not been investigated in these studies: partner choice in the surrounding market^2^,^3^. If subordinates lack options to switch groups or trade with other dominants, or if dominants have no outside options for recruiting helpers, there is limited scope for competition for partners^2^,^3^. In the absence of outside options, the only alternative to trading with specific partners is not trading at all: defection by a subordinate, or its eviction by the dominant. Indeed, with notable exceptions^9^,^11^,^19^, theoretical models of helping behaviour in cooperative breeders typically assume that group members have a choice between breeding alone or belonging to only one particular group^10^,^14^,^20^,^21^,^22^.

The critical experiments to test whether market forces affect the exchange of commodities in cooperative breeders are first, to test whether there is partner choice; and second, to manipulate the outside options available, altering competition for trading partners^10^,^23^. We carried out both tests using cooperatively breeding groups of the paper wasp *Polistes dominula* (Fig. 1a,b). In early spring, mated *P. dominula* females emerge from hibernation and initiate nests in groups of typically fewer than 10 individuals of the same generation^24^. Solitary breeding occurs at a low rate in our study population in southern Spain (∼6.4% of females initiated nests alone in Zanette and Field^24^), but the risk of nest failure is greater for solitary breeders than for groups^14^,^24^. Because groups form synchronously at our field site, with thousands of females founding nests simultaneously along stretches of a few hundred metres of cactus hedge (*Opuntia ficus-indica*), females potentially have a choice of cooperative partners. Once a nest has been initiated, additional females may join, and nest residents form a linear dominance hierarchy where one becomes the dominant breeder producing most or all of the offspring, while the remaining residents become subordinates that help build the nest and care for the dominant's brood^14^. Nest residents are often sisters, but a significant fraction of subordinates are genetically unrelated to the dominant breeder they are helping^25^,^26^. Subordinates may gain direct fitness through occasional egg laying, or through nest inheritance^14^: if the dominant dies, the highest ranking subordinate then inherits the breeding position^27^. The first offspring that mature during late spring become workers that help to rear more workers, and eventually new reproductives that mate and overwinter. We focus here on the pre-worker phase, when females from the same generation live as cooperative breeders.

In *P. dominula*, the dominant female can adjust her reproductive output according to group size. More offspring are produced when more subordinate helpers are present^14^,^25^, and if the number of helpers decreases unexpectedly, excess offspring can be recycled at minimal cost by feeding eggs and small larvae to larger, more valuable larvae^28^. This means that each additional subordinate has extra value to the dominant, and that any investment provided by helpers will translate to higher reproductive output, even if the helper defects before offspring maturation^28^. This differs from most cooperatively breeding vertebrate systems, where reproductive output cannot so easily be adjusted to fluctuations in help available, and where previous investment may be lost if helpers defect^28^,^29^. It is also important to note that trader classes are not entirely fixed in *P. dominula*, because subordinates can become dominants if, for example, they leave to initiate a new nest alone or with others; and dominants can lose their breeding position to a subordinate or joiner that challenges them^26^. In total, this flexibility means that the effect of simply altering the ratio of trader classes within groups, for example, by reducing the number of subordinates, may be transient and may not result in dominants paying more for help or subordinates paying less for group membership^15^,^16^,^17^. Taken together, these points suggest that the supply of outside options will have the biggest effect on the exchange of cooperative behaviours within *P. dominula* social groups.

We first tested and confirmed that both new and established nest residents do in fact have a choice of cooperative partners (see Results), as required for biological market theory to apply. Given that females do have multiple options, the theory predicts that supply of and demand for those options will influence not only partner choice, but also behaviour within groups^2^,^6^,^11^. We therefore next manipulated the outside options available to nest residents, to test whether market forces influence a key aspect of helping behaviour: foraging effort. Foraging is costly for subordinates: it correlates positively with individual mortality^27^, and will therefore reduce a subordinate's chance of eventually inheriting the breeding position^30^. For this reason, and because subordinates are less closely related to group offspring than dominants, the two trading classes have different interests, with dominants preferring to receive a higher payment for group membership than subordinates prefer to pay^27^. The deal settled on will depend on the relative demand for helping behaviour versus group membership^11^.

We assume that helping behaviour is in highest demand in *P. dominula*, so that dominant breeders essentially compete to retain their subordinates and to recruit additional helpers. We assume this for several reasons: (i) dominants always benefit from recruiting more helpers, because both reproductive output and group survival increase with group size^14^; (ii) when a dominant recruits an additional helper, she does not simultaneously reject an existing partner: there is no replacement of partners, as classical market theory usually assumes^2^; (iii) subordinates retain the option of initiating new nests, alone or with others, and so have other options beyond trading with established dominants. Binding agreements about future behaviour are likely unnecessary for trading to occur in this system^4^: first, a dominant cannot easily accept help from a subordinate then later renege on the inheritance payoff that the subordinate may receive after the dominant's death^31^; second, although it is in the interest of a subordinate to leave if a higher-payoff option becomes available, any investment she makes before defecting still increases reproductive output for the dominant^28^.

To summarize our main findings: our first result is that females have a choice of partners when they form social groups, confirming the potential for a market in this species. Our second result is that increasing the availability of outside options for subordinates leads to them decreasing their foraging effort within groups. These results are in accordance with predictions from biological market theory: that increased competition among dominants for cooperative partners will induce them to accept a lower price for group membership. Our findings imply that it is necessary to take market forces and specifically outside options into consideration when predicting the amount of help provided in cooperative societies.

## Results

### Partner choice experiment

To test whether a new joiner had a choice of nests to potentially join, we waited until a new female joined each of 32 established groups in the field, and then permanently removed both the nest and all of its original residents, releasing just the joiner. Similarly, to test whether established subordinates have the option of leaving to join other groups, we removed a further 34 nests and their residents, releasing just one low ranking subordinate from each group. We found that at least 24 of the 32 released new joiners and 17 of the 34 released subordinates subsequently joined other established nests in the population, or initiated new nests with others. Only a single new joiner and a single subordinate started nesting alone. This finding demonstrates that whether they are new or established nest residents, most females have more than one choice of partner, confirming the potential for a biological market in this species^2^,^3^. It further suggests that alternative partners include nest-less floaters that can be recruited to co-initiate new nests, as well as residents on other established nests.

### Market manipulation experiment

**Figure 1:**
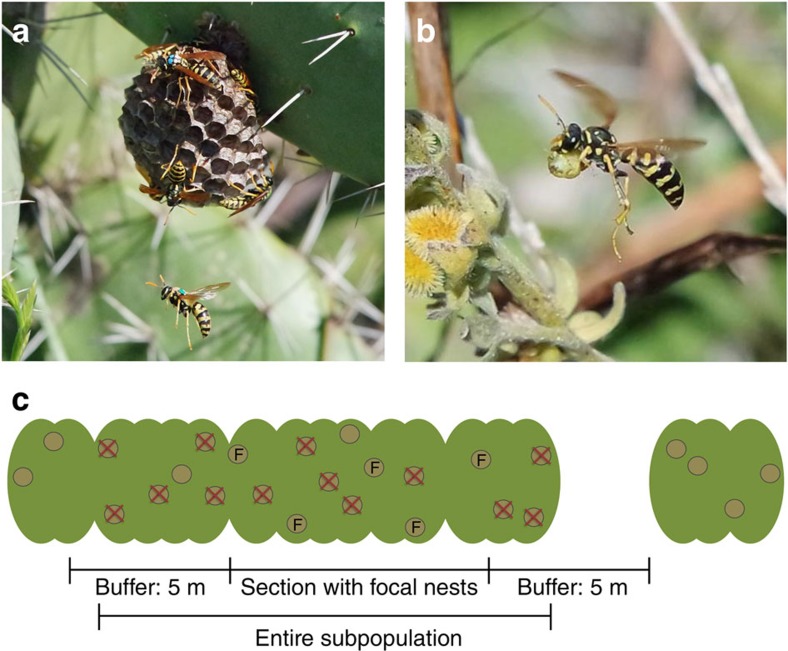
The paper wasp *Polistes dominula* at our field site in Spain. (**a**) A nest resident returning to her nest. (**b**) A female carrying a food ball. (**c**) Schematic drawing showing a subpopulation of wasp nests on a section of cactus hedge used in the Market Manipulation experiment: focal, un-manipulated nests are circles marked with an ‘F' (∼25% of the nests within a section), while the rest were termed ‘market nests'. The majority of market nests were removed in the Nest Removal and Partner Release treatments (indicated by an ‘X'). Five meter buffer zones on each side of the cactus section did not contain focal nests but received the same treatment as the rest of the section. Buffer zones sometimes included areas with no nests, as shown on the right. Photos courtesy of Tanya Pennell.

To manipulate the surrounding market, we altered the supply of outside options available to the two trader classes without modifying the natural ratio of subordinates to dominants within groups. Specifically, we imposed one of three treatments within each of nine *P. dominula* subpopulations (three subpopulations/treatment; Fig. 1c; Table 1) and recorded subordinate foraging effort before and after treatment on a total of 43 un-manipulated focal nests: (i) Control (C) where the supply of outside options was not manipulated; (ii) Nest Removal (NR) treatment where we permanently removed 50–75% of nests and all of their residents. This treatment freed up nesting spots, potentially suitable for subordinates to initiate new nests alone or with others. We therefore predicted that subordinate foraging effort would decrease following the treatment compared with Controls: the greater availability of outside options for subordinates should effectively increase competition among dominants, inducing them to accept a lower price for group membership; (iii) Partner Release (PR) treatment where we again removed up to 75% of nests, but coupled this with releasing one randomly chosen individual from each nest removed (henceforth these nest-less females are termed ‘floaters'). The expected outcome of Partner Release depended on whether floaters primarily represented potential partners for focal subordinates to co-initiate new nests; or whether they represented potential partners for focal dominants. In the former case, Partner Release would further improve the outside options for subordinates, so that competition among dominants would intensify relative to Nest Removal, and we would predict that subordinate work effort would be even lower. Alternatively, if floaters were primarily recruited by dominants as helpers, competition among dominants would be reduced, and we predict that subordinate helping effort would increase relative to Nest Removal. Finally, if the supply of outside options does not influence cooperative behaviour in *P. dominula*, we would observe no difference in subordinate foraging effort between treatments. This might occur if nest residents lack information about the outside options available, and would suggest there is no biological market in this system.

**Figure 2:**
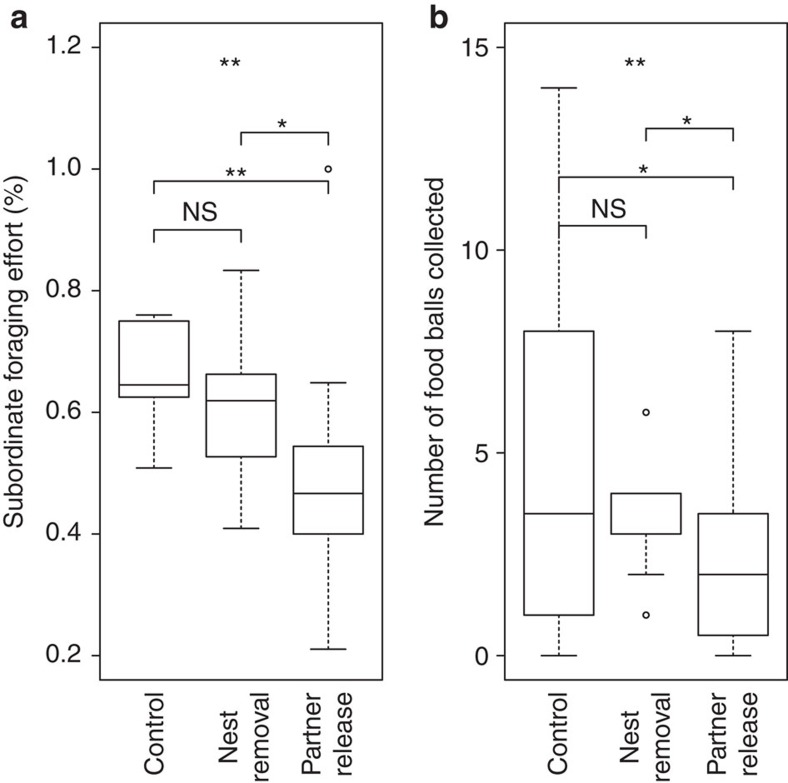
Subordinate foraging after the Market Manipulation treatments. The effect of treatment on (**a**) collective subordinate foraging effort per nest, measured as proportion of observations where individuals were absent from the nest in daytime censuses post-treatment (GLMM: overall effect of treatment: *P*=0.0023; *N*=43; differences between treatments: PR and C: *P*=0.0011; NR and PR: *P*=0.021; NR and C: *P*=0.23. (**b**) Number of food balls collected per nest in 5 h videos post-treatment (GLMM: overall effect of treatment: *P*=0.0037; *N*=43; difference between treatments: PR and C: *P*=0.011; NR and PR: *P*=0.039; NR and C: *P*=0.14). Boxes represent second and third quartiles with the median value indicated as a black line; whiskers stretch from lower to upper maximum values, except from outliers indicated as dots. Stars indicate significance levels from GLMMs: ‘*' indicates *P*<0.05; ‘**' indicates *P*<0.01

Manipulating outside options clearly did influence the cooperative behaviour of residents on focal nests. Compared with Controls, subordinates foraged significantly less following the Partner Release treatment, where surrounding nests and their residents were removed and floaters were released. Foraging effort was also lower following Nest Removal than in Control, but this difference was not significant (generalized linear mixed model (GLMM): overall effect of treatment: *X*^2^=12.13, *P*=0.0023, block, group size and average relatedness: *X*^2^<2, *P*>0.05; effect of treatment in *post hoc* tests: PR and C: *X*^2^=10.63, *P*=0.0011; NR and PR: *X*^2^=5.31, *P*=0.021; NR and C: *X*^2^=1.44, *P*=0.23; Fig. 2a). These results are consistent with the idea that floaters represent potential partners for subordinates to co-initiate nests, so that Partner Release increased competition among dominants for helpers. Supporting this hypothesis, floaters in Partner Release were more likely to initiate new nests than they were to join focal nests (18 and 9 floaters did so respectively). In video recordings, subordinate foraging effort at the individual level was positively associated with how much aggression a wasp received while on the nest both before treatment (GLMM: effect of aggression received: *X*^2^=18.21, *P*<0.001, block: *X*^2^=15.84, *P*<0.001, treatment and brood value: *X*^2^<2, *P*>0.05) and after treatment (GLMM: effect of aggression received: *X*^2^=8.18, *P*=0.0042, block: *X*^2^=6.46, *P*=0.040, treatment: *X*^2^=5.14, *P*=0.077, brood value: *X*^2^=9.79, *P*=0.0018). However, as foraging effort and inheritance rank in the social hierarchy correlate, it is unclear whether aggression serves mainly to reinforce an individual's position in the hierarchy^32^ or to enforce foraging behaviour^33^.

Reduced foraging effort in Nest Removal and Partner Release treatments was accompanied by the collection of significantly fewer food balls (GLMM: overall effect of treatment: *z*=−2.90, *P*=0.0037, group size: *z*=2.40, *P*=0.016, block and brood value: *z*<1, *P*>0.05; *post hoc* tests: PR and C: treatment: *z*=−2.54, *P*=0.011, group size: *z*=2.43, *P*=0.015; NR and PR: treatment: *z*=−2.07, *P*=0.039, group size: *z*=1.20, *P*=0.23; NR and C: treatment: *z*=−1.46, *P*=0.14, group size: *z*=2.46, *P*=0.014; Fig. 2b). Although dominants would therefore receive less help with raising current offspring by accepting a lower price for group membership, this cost might be counterbalanced by the benefits of inducing current subordinates to stay in the group, and through recruiting joiners as additional helpers. Indeed, more focal nests received joiners after Partner Release (42%) than after the Control (15%) or Nest Removal (17%) treatments (although this difference was not significant: *X*^2^=4.75, *P*=0.093), and treatment had no overall effect on nesting success (in generalized linear models (GLMs) accounting for block, groups size, average relatedness and brood value, there was no effect (*P*>0.35) of treatment on date of worker emergence, brood developmental stage at worker emergence or brood growth rate).

While nest density was positively associated with foraging effort after treatment (GLMM: *X*^2^=3.87, *P*=0.049), it did not predict foraging effort before treatment (GLMM: *X*^2^=1.08, *P*=0.30). Similarly, nest density was positively associated with number of food balls collected after treatment (GLMM: treatment: *z*=3.19, *P*=0.0014, block, group size and brood value: *z*<2, *P*>0.05) but not before treatment (GLMM: treatment: *z*=0.70, *P*=0.49, brood value: *z*=2.36, *P*=0.019, group size: *z*=−0.12, *P*=0.91). We discuss below why changes in nest density following our treatments are unlikely to be the explanation for our main results.

### The size of the market

Our data suggest that the market for cooperative partners is large in *P. dominula*: females could nest with both close relatives and non-relatives, and therefore may have many alternative partners available. After the Market Manipulation treatment, five females joined focal groups containing no close relatives (sisters), while six joined groups with sisters. Furthermore, 167 out of all 1,351 females in the treated subpopulations had no sisters within their groups, despite having up to 25 sisters in other groups; and the 225 wasps that had no sisters in their entire subpopulation were not forced to nest alone—only 10 did so. The number of nests with sisters in the market did not predict individual subordinate foraging effort (GLMM: full model: number of nests with sisters, relatedness to the dominant and group size, *X*^2^=3.99, *P*=0.41), suggesting that it was not the reduced number of close relatives specifically that caused the treatment effect on foraging effort.

## Discussion

Here we document the presence of partner choice in the cooperatively breeding paper wasp *P. dominula*, and demonstrate that the supply of outside options affects helping behaviour within un-manipulated social groups in the field. These results support the hypothesis that there is a biological market in *P. dominula*. Other studies have found that the ratio of subordinates to dominants correlates with the value of grooming within cooperative mammal groups^15^,^16^,^17^, but until now no study has manipulated the outside options available to trading partners in natural settings. In biological markets, trader classes are often fixed, as in the inter-specific trade between cleaner fish and their clients^6^,^34^. In such markets, the supply and demand of commodities can be manipulated by simply altering the ratio of the two trader classes^2^. In cooperative breeders, however, individuals can switch between trader classes, so that the ratio could re-adjust following such manipulations. We therefore instead manipulated the ratio of outside options available to the two trader classes, rather than the ratio of dominants to subordinates itself.

In *P. dominula*, dominants benefit by recruiting more helpers, and by enticing established subordinates to stay and help, while subordinates have an incentive to explore other options. Initiating a new nest is a high-risk/high-reward option for a subordinate that could lead to a higher social rank, or even dominant breeding status. When we freed up suitable nesting spots, we likely increased the number of alternative options available to subordinates relative to dominants. However, without a large choice of available nesting partners, the incentive for subordinates to leave their current nests might still be relatively low: initiating a nest alone is rarely successful^14^,^28^. This lack of available partners could explain why we saw only a small, non-significant effect of the Nest Removal treatment on subordinate foraging effort (Fig. 2a,b). However, when we coupled the increase in supply of suitable nesting spots with the release of potential nesting partners we further improved the alternative options for subordinates, and this led to a significant decrease in foraging effort in the Partner Release treatment. Similarly, in a laboratory study of cooperatively breeding cichlids, *Neolamprologus pulcher*, where subordinate helpers ‘pay-to-stay'^35^,^36^, helpers were presented with outside options for independent breeding^37^. Larger subordinates, which are more likely to become independent breeders in the future, subsequently reduced their submissive behaviour towards dominant breeders^37^, suggesting that dominants may have had to accept a worse deal in order to retain their subordinates.

Our data do not support an alternative interpretation of our results: that subordinate foraging effort fell in Nest Removal and Partner Release treatments because we reduced local nest density, thereby reducing competition for prey in the local environment. Nest density was not associated with foraging effort before our manipulation, and even though nest density was reduced to the same extent in Nest Removal and Partner Release, foraging effort differed significantly following the two treatments (Fig. 2a). Furthermore, reduced foraging effort led to subordinates collecting fewer food balls than in the Control treatment, rather than to less effort being required to collect the same numbers of food balls. Another possible explanation for our results could be that subordinates interpreted the disappearance of neighbouring nests as an indication of higher predation risk (Nest Removal and Partner Release), and foraged less in order to stay and defend the nest against predators. This perception might have been further heightened when they additionally encountered more floaters than usual (Partner Release). However, we find this explanation unlikely, because there is no evidence that a larger number of nest residents is more effective in predator defence in this^28^ and other paper wasp species^38^,^39^. Additionally, subordinates in a related paper wasp are less likely than the dominant breeder to defend the nest against both predators and conspecific nest usurpers (*Polistes fuscatus*^40^,^41^).

Our study demonstrates that the supply of outside options influences helping behaviour in a large-scale field study of a cooperative breeder. The pay-to-stay hypothesis, where subordinates are assumed to pay for group membership with work effort^12^,^13^, has received empirical support in cooperatively breeding cichlid fishes, where subordinates may be evicted from the group if they do not pay by helping^36^. However, this hypothesis lacks key market concepts such as competition for cooperative partners and the idea that supply and demand determine trade values. Our results suggest that partner choice and outside options do influence cooperative behaviour in groups of paper wasps. They further suggest that the market may be large, since individuals could join nests with either genetic relatives or unrelated individuals. These findings imply that in order to predict the level of help provided by a subordinate, it is necessary to take into account the state of the surrounding market, not just within-group variables such as the subordinate's social rank and her genetic relatedness to other group members.

A question for the future will be to investigate how helping behaviour is regulated. We have referred to dominants and subordinates as the trading classes in our study system. However, work effort might be enforced by high-ranking subordinates^33^ rather than by dominants^42^ as each nest resident directs aggression mainly towards the individual ranked immediately below it^32^. In our study, foraging effort was positively associated with the amount of aggression a subordinate received, but it is currently unclear whether aggression functions primarily to maintain the aggressor's position in the hierarchy or to directly enforce foraging^32^,^43^. Another question concerns how females assess the state of the market. Both dominants and subordinates were seen visiting other nests, but the precise information they obtained about outside options remains unknown. Our results suggest that biological market effects influence the level of helping in wasp societies, and future research should aim to elucidate the mechanisms underlying this.

## Methods

### Study organism and handling of animals

*Polistes dominula* is a primitively eusocial wasp (lacking morphological castes) found in most of Europe, Northern Africa and parts of Asia and North America. In our population, pre-mated females from the same generation found nests alone or in small groups in early spring, after overwintering. Female offspring maturing in late spring become workers; those maturing during summer overwinter to found nests the following year^14^. Field studies were conducted prior to worker maturation during March to May 2013 (Partner Choice experiment) and 2014 (Partner Choice and Market Manipulation experiments) at a rural field site near Conil de la Frontera, Cadiz, Spain (N 36°15′9.286″ W 6°3′52.616″) (refs 14, 27). Here wasp nests are abundant on long, straight hedges of prickly pear cactus (*Opuntia ficus-indica*) that divide up small arable/pasture fields.

All nests involved in the experiments (Partner Choice: *N*∼700; Market Manipulation: *N*∼300) were tagged and numbered. For each nest, the distance along the cactus hedge and height above ground were measured to the nearest 5 cm. Nest residents (females in focal nests in the Partner Choice experiment: *N*∼350; females from all nests in the Market Manipulation experiment: *N*∼1,500) were collected before sunrise (6:00–7:00), transported to the laboratory and given individual paint marks on the thorax using enamel paints. Each female from a focal nest (in either experiment) was given a unique code of four coloured dots, and each female from a ‘market nest' (only for the Market Manipulation experiment; see below) was given a nest-specific colour code of two dots. DNA samples were obtained by cutting the tarsus from a middle leg at the time of marking^14^. Tarsus samples were kept in 100% ethanol at ∼4 °C until used for genotyping. After marking and DNA sampling, wasps were kept at ∼4 °C until being released close to their nests on the same morning as collection, before 11:00. When nests and nest residents were permanently removed (as a part of an experimental treatment), they were released at a field site 2.5 km away: none returned to their original site.

### Partner choice experimental procedure

Before the manipulation, we carried out daytime censusing (see below) on focal nests to obtain information about each individual's rank in the hierarchy. We also monitored the arrival of new joiners by censusing nests every 2–3 days in the evening (18:00–20:00) when all residents have returned to their nests. In 32 nests we observed a natural joining event and successfully applied treatment as follows: two days after joining we marked the joiner and two days later in the morning before sunrise (6:00–8:00) we removed the nest and all nest residents and immediately released only the new joiner. If some known nest residents were absent at the time of treatment, we left the nest *in situ* for a maximum of 48 additional hours before removing it, allowing us to attract and remove remaining residents. In 34 additional groups we chose a low ranking established subordinate and performed the same treatment: we removed the nest and all residents but released the chosen subordinate. During the following 4–8 weeks, we searched for the released joiners and subordinates by censusing all nests in the population every 2–4 days both during daytime and in the evenings, and by locating newly initiated nests.

### Market manipulation experimental setup

For each replicate, three spatially adjacent sections of cactus of about equal length were chosen within an experimental block of cactus hedge (Fig. 1c; Table 1). In order to test for potential confounding effects of nest density, we replicated the three treatments within three blocks of cactus hedge that varied naturally in nest density. Each section (each containing one of our nine wasp subpopulations) within a block was assigned one of three treatments: Control, Nest Removal or Partner Release (Table 1). Within each section ∼25% of the nests were chosen as focal nests, aiming to have an even spatial spread within the section. The remaining nests were deemed ‘market nests' (Fig. 1c). We included a 5 m buffer zone at either end of each section, wherein all nests were deemed market nests and received the same treatment as other market nests. Buffer zones were included to prevent different treatments within a block from influencing each other; previous studies show that wasps visit and switch nests mainly within a 5 m radius^24^. We refer to wasps within each experimental cactus section as a ‘subpopulation'. Both before and after treatment we estimated: (i) Foraging effort of subordinates, measured as proportion of time spent off the nest foraging, based on daytime censuses and video recordings (5 h pre- and 5 h post-treatment); time spent off the nest was a good measure of foraging effort: it positively correlated with the number of food balls collected (*N*=118 subordinates; Spearman's rank correlation: *P*=0.010, *rho*=0.24); (ii) Within-nest aggression and foraging returns from video recordings, and (iii) Brood development using field censuses at ∼10-day intervals. Further details about these measurements are given below. After treatment we also obtained the following measures for each nest: (i) date of worker maturation, (ii) nest failure prior to worker maturation, and (iii) whether nests received joiners after treatment, before worker maturation. We had a total of 61 focal nests across all nine subpopulations. Nine of these failed after treatment before we could obtain all of the post-treatment data, and another nine were nests occupied by solitary females that were left out of the main analyses. Hence, 43 focal nests were used in most statistical analyses (Table 1).

### Market manipulation treatment

Before sunrise (5:00–8:00), we permanently removed all market nests (including market nests in buffer zones) and their residents in Nest Removal and Partner Release treatment sections (Fig. 1c). In Partner Release sections we further released one randomly chosen resident from each removed nest back into its subpopulation. If some nest residents were absent from a market nest at the time of treatment, we left the nest for a maximum of 48 additional hours before removing it, allowing us to attract and remove remaining residents. Some market nests were impossible to reach and were left untouched (0–10 nests per section; Table 1). All market nests in Control sections were un-manipulated. Focal nests in all treatments were censused at the time of treatment to determine which residents were present, but were otherwise un-manipulated and were then left undisturbed for two days before daytime census was resumed. We successfully removed 50–75% of all nests (calculation including focal nests) in Nest Removal and Partner Release sections, except in Nest Removal second block (Table 1). This section experienced high nest failure pre-treatment and so only two nests were removed upon treatment. This section may be viewed as a failed treatment, but main statistical analyses gave the same qualitative result whether performed on the full data set or on a subset of the data excluding the two focal nests in this section.

### Daytime and brood census

Daytime census consisted of spot-checks, recording which nest residents were present on the nest on sunny days during the main foraging period (11.00–17.00). Nests were censused 3–5 times per day (minimum 30 min between censuses) every 3–4 days for 14 days (±2 days) before treatment in the Market Manipulation experiment, and from after treatment until worker emergence (10–39 days, median=22.5). Individuals were ranked according to how much time they spent on the nest, with rank 1 (the dominant) spending the most time and the lowest rank spending the least time on the nest^27^.

Foraging effort in the Market Manipulation experiment was estimated as the overall proportion of censuses during which a subordinate was absent from the nest. Dominants rarely forage and were excluded from all analyses of foraging effort. If there was any doubt about dominant identity on a nest, the two females that spent the most time on the nest were both considered dominants and excluded from analyses. We also excluded any nest that was occupied by just a single female, either before or after treatment, since such nests contained no subordinates.

Brood censuses were performed 1–2 days before treatment in the Market Manipulation experiment, 5–7 days after treatment and then at 10–11 day intervals until worker emergence. They involved counting brood at different developmental stages and allocating a brood value to the nest based on the following scores: small larva (given a value of 1.5), medium larva (2), large larva (3) and pupa (4); a cell without a larva or pupa was assumed to contain an egg (1). They also involved determining which nest residents were present on the nest before sunrise. This was the most reliable method of tracking group membership, because subordinates that forage most of the time might be completely missed during daytime censuses.

### Video data

We video recorded each focal nest in the Market Manipulation experiment for 5 h on sunny days (between 11:00 and 17:00), 1–4 days (median=1) before the treatment day and again 5–10 days (median=5) after treatment. Data were extracted from each video (*N*=86) by one of seven people, all trained in the same way by one person, who spot-checked for consistency. All behavioural interactions between nest residents were recorded and given values according to the level of aggressiveness: antennation (given a value of 1), food sharing (2) or aggression (3; including all more aggressive encounters such as bite, chew and lunge). For each nest we calculated a mean per-female aggression value by summing aggression values and dividing by the number of nest resident (dominants included). All residents returning to the nest were recorded as bringing back either nothing visible, liquid food (evidenced by trophallaxing) or a solid food ball.

### Genotyping and relatedness

Protocols were similar to those described previously^14^,^30^. Following DNA extraction from tarsus samples, samples were genotyped at nine microsatellite loci used previously in studies of the same population^14^,^30^,^44^,^45^. Each locus had between 7 and 49 different alleles in our samples (median=15). All loci were amplified in a single multiplex reaction using the Qiagen multiplex PCR kit (Qiagen, Venlo, The Netherlands). The reaction contained 10–100 ng template DNA, 1 μl of 2 × Multiplex master mix (3 mM MgCl_2_) and 1 μl of primer mix. The primer mix consisted of 0.375 μmol each of Pdom7 and Pdom127b, 0.4375 μmol of Pdom25 and Pbe128TAG, 0.5 μmol each of Pdom2 and Pdom140, 0.625 μmol Pdom20, 0.9375 μmol Pdom1 and 3.5625 μmol Pdom122. A drop of mineral oil was added to prevent evaporation. PCR was performed in a G-storm GS2 thermal cycler with a temperature profile of 95 °C for 15 min; 35 cycles of 94 °C for 30 s, 57 °C for 90 s and 72 °C for 60 s; followed by a final extension step of 60 °C for 30 min. Each plate included a positive and negative control to check for consistency of amplification. Following dilution 135 fold with water, GeneScan LIZ 500 size standard was added (Applied Biosystems). Allele size was determined using a 48-well capillary 3730 Sequencer (Applied Biosystems) and alleles were called using GeneMapper 3.7 (Applied Biosystems). Tests for linkage disequilibrium, null alleles and deviations from Hardy-Weinberg equilibrium revealed no significant deviations from chance expectations for these loci^14^. Relatedness 5.0.8 software^46^ was used to calculate average relatedness between nest residents. The Full Sibship Reconstruction procedure in Kingroup v2 software^30^,^47^ was used to identify groups of sisters within each block (primary hypothesis: haplodiploid sisters; null hypothesis: haplodiploid cousins)^14^. We then counted the number of sisters each resident had in its own nest, the number of sisters it had in other nests and the number of nests (other than its own) that contained sisters. Only individuals with at least 6 out of 9 loci scored successfully were used (median number of successful loci per sample=9); 1,452 out of 1,509 wasps were successfully genotyped.

### Statistics

The effect of treatment on response variables was tested with GLMs or GLMMs (packages: lme4 (ref. 48) and glmmADMB (ref. 49)) using the statistical software R (ref. 50), with individual nests as data points (*N*=43). All models were tested for collinearity among predictor variables, and for models with a Gaussian error structure we ensured that residuals were normally distributed and homogenous and transformed variables accordingly. For models with Poisson or Binomial error structure we tested for overdispersion and corrected for it in the following way: if Poisson models were overdispersed we instead used a Negative Binomial error structure^49^, and if Binomial models were overdispersed we added a random effect of nest ID (ref. 51).

In all models, non-significant predictors (*P*>0.05) were removed to obtain more accurate *P* values for the remaining predictors. *P* values from GLMMs were obtained by model comparisons (*χ*^2^), as were *P* values of categorical variables in GLMs.

Each hypothesis about the effect of treatment or nest density on foraging effort or food returns was tested by building three full models: one as a control performed on pre-treatment data where treatment was expected to show no effect (the results of which are not reported as they confirmed there was never an effect of treatment prior to treatment day); a second model using post-treatment data to test for an effect of treatment (the results of which are reported); and a third model using the difference between before and after treatment reflecting the change to focal nests induced by the application of treatment (the results of which are not reported, as they all showed qualitatively similar results to the post-treatment analyses).

When treatment significantly predicted a response variable in a main model (including data from all three treatments), *post hoc* tests were performed to investigate the differences between each treatment-pair by running a model similar to the main model on three subsets of the data comparing a pair of treatments at a time. These *post hoc* models included only predictors that showed significant effects in the main model.

To test for the possibility that nest density, rather than the intended market effect, may have caused observed results, we ran similar models to the main models where we replaced treatment with nest density (calculated across the entire sections including buffer zones, Fig. 1c) as a predictor. In these density models we additionally included block as a predictor (except in pre-treatment models, since block here correlated with nest density; Table 1), as well as any predictors that showed a significant effect in the main models.

All main models were also run on a subset of the data where the two Nest Removal second block nests were excluded, to ensure that these did not skew the results (Table 1). None of the results from these models differed qualitatively from those of the main models and so are not reported.

Models testing the effect of treatment on foraging effort (as estimated in daytime censuses) had as response variable a binomial vector of the number of times subordinates were absent versus present on the nest (representing the proportion of observations where subordinates were absent from the nest, *N*=43). These logistic regression GLMMs had Binomial error structure, with nest ID included as a random effect to eliminate overdispersion^51^. Predictor variables included treatment, block, number of residents (because per capita foraging effort may be affected by group size) and average relatedness among nest residents (because within-nest relatedness may influence how hard a subordinate will work).

Additional GLMMs were built testing whether individual foraging effort was influenced by the number of nests with sisters available in the market. These models were run with individual subordinates as data points (subordinates only, *N*=88) and included nest ID as a random effect. Predictors included number of nests with sisters, relatedness to the dominant, the interaction between the two (to allow for the market to have different effects on foraging effort depending on the relatedness to the dominant) and number of nest residents. Models had Binomial errors and individual wasp ID was included as an additional random effect to eliminate overdispersion.

To test for the effect of treatment on food returns in video recordings, we used the total number of food balls collected per nest (range: 0–14, median=3) as a response variable in GLMMs with Negative Binomial error (*N*=43). We included as predictors: treatment, block, number of residents and brood value at the time of videoing (because video recordings were snapshots in time and behaviours were expected to be influenced by immediate state such as developmental state of the brood) and as a random effect the name of the person watching the video.

We tested whether the amount of aggression received by other group members while on the nest (as observed from video recordings) affected foraging effort at the individual level (only subordinates included; *N*=107). In these GLMMs with Gaussian error we used proportion of time spent absent from the nest per subordinate as response variable, and included log(aggression value received per hour+1), treatment, block and brood value as predictors, and nest ID and the name of the person watching the video as random effects.

For each of the following response variables, one GLM was built testing the effect of treatment on nest success variables: Date of worker emergence (Gaussian error), brood value at worker emergence (square root transformed, Gaussian error), brood developmental rate (average brood value gained per day for three weeks after treatment, Gaussian error) and nest failure (Binomial error). In all of these models, the following predictors were included: treatment, block, number of residents, average relatedness and pre-treatment brood value to account for initial differences among nests.

### Data availability

All raw data are available online via Dryad Digital Repository ( http://dx.doi.org/10.5061/dryad.87hm1).

## Additional information

**How to cite this article:** Grinsted, L. & Field, J. Market forces influence helping behaviour in cooperatively breeding paper wasps. *Nat. Commun.* **8,** 13750 doi: 10.1038/ncomms13750 (2017).

**Publisher's note**: Springer Nature remains neutral with regard to jurisdictional claims in published maps and institutional affiliations.

## Figures and Tables

**Table 1:** Overview of treated subpopulations.

**Block**	Treatment	Number of nests in the section, incl. buffers	Number of successful focal nests	Number of nests removed upon treatment	Proportion of nests removed upon treatment (%)	Number of new nests built after treatment	Number of joiners to focal nests after treatment	Length of section incl. buffers (m)	Nest density before treatment, incl. buffers (nests m^**−1**^**)**	Nest density after treatment, incl. buffers (nests m^**−1**^**)**
1st	Control	54	7	0	NA	1	1	15.9	3.41	3.47
High density;	Nest Removal	60	6	42	70.0	1	3	15.2	3.95	1.32
Treated on 12 April 2014	Partner Release	46	6	34	73.9	6	4	16.4	2.80	1.22
2nd	Control	22	4	0	NA	0	3	14.0	1.57	1.57
Low density; Treated	Nest Removal	5	2	2	40.0	0	0	5.0	1.00	0.60
on 24 April 2014	Partner Release	14	4	8	57.1	0	1	10.7	1.31	0.56
3rd	Control	33	3	0	NA	0	0	16.1	2.05	2.05
Medium density;	Nest Removal	28	6	14	50.0	0	0	16.3	1.72	0.80
Treated on 28 April 2014	Partner Release	28	5	21	75.0	3	4	16.4	1.71	0.61

Overview of number of nests, nest densities and other parameters in each of the nine treated cactus sections: three treatment sections within each of three blocks. Nest Removal 2nd Block is written in grey indicating that treatment in this section may be viewed as failed.
